# Comparison of occlusal caries detection using the ICDAS criteria on extracted teeth or their photographs

**DOI:** 10.1186/s12903-016-0291-z

**Published:** 2016-09-07

**Authors:** P. Bottenberg, W. Jacquet, C. Behrens, V. Stachniss, A. Jablonski-Momeni

**Affiliations:** 1 Department of Oral Health Sciences, Vrije Universiteit Brussel, Laarbeeklaan 103, 1090 Brussels, Belgium; 2 Depatment of Educational Sciences EDWE-LOCI, Vrije Universiteit Brussel, Pleinlaan 2, 1050 Ixelles, Brussels, Belgium; 3 Dental School, Department of Pediatric and Community Dentistry, Philipps University of Marburg, Marburg, Germany; 4 Dental School, Department of Restorative Dentistry, Philipps University of Marburg, Georg-Voigt-Straße 3, D-35039 Marburg, Germany

**Keywords:** Caries detection, Occlusal caries, ICDAS, Digital images, ROC curves

## Abstract

**Background:**

Using photographs of occlusal surfaces instead of extracted teeth for the detection of caries can be useful in multicenter studies or education. Using a panel of observers, ICDAS scores on teeth or photographs were evaluated against the histological gold standard. The hypothesis was that both outcomes were equivalent.

**Methods:**

Four examiners with different experience in ICDAS scored photographs of occlusal surfaces of 100 extracted teeth on a monitor using ICDAS criteria. Two of the examiners had previously scored extracted teeth prior to photography. Digital images of histological sections of the teeth were observed by all examiners and consensus scores were given for each investigation site (gold standard). Kappa statistics and Spearman correlation coefficients as well as repeated measure ANOVA were performed. ROC curves were constructed for each examiner and the areas under the ROC-curves (AUC) of both scoring techniques (extracted teeth, digital images) were compared (α = 0.05).

**Results:**

Intra- and inter-rater kappa for ICDAS on teeth were 0.81–0.94 and on photographs 0.54–0.88, respectively. Correlation with histology was 0.58– 0.61 for the teeth and 0.50–0.62 for the photographs. AUC of ICDAS scores of extracted teeth (mean 0.89) were slightly higher than those for photographs (mean 0.84). However, both AUC values were not statistically significant (*p* = 0.38).

**Conclusion:**

Using photographs to assess occlusal surfaces with the ICDAS criteria was not statistically different from scoring the extracted teeth.

## Background

Visual-tactile caries detection remains a process in which a human observer judges differences in color, texture, transparency or radiolucency to come to a conclusion about the presence and extent of pathological processes under the tooth surface. Until the advent of methods which automatically and directly detect demineralization in a reliable and affordable way, caries detection relies on the clinician as final signal processing tool. Each individual observer is liable to have a different interpretation of these signals based on visual acuity, neurological signal processing, experience or education.

The well-established ICDAS (“International Caries Detection and Assessment System”) visual caries detection system enables lesions at various stages to be clinically detected [1]. ICDAS was introduced to standardize the interpretation of the visual signs of caries detection. Besides the score of “0” (caries free), six different stages of lesion severity are registered. This method exhibits good reproducibility and a clinically acceptable specificity and sensitivity to the detection of occlusal caries [2].

However, as with all methods, observers have to be trained in using ICDAS correctly. This is logistically demanding when ICDAS is to be used in other settings than small groups of intensively calibrated and trained observers as was the practice in scientific reports hitherto. The logistical challenge is much greater in larger groups such as dentists with different backgrounds and experience participating in epidemiologic surveys or training of graduate students.

Moreover, organizing multi-center studies necessitates travels of trainers and examiners to perform the examinations. The logistics involved generally limited the number of observers.

However, it is preferable to use the same set of teeth in order to compare all participants. Physically sending teeth to different centers can lead to damage or bias due to desiccation of the samples. This also limits the repetitive use of extracted teeth in student education.

When calibration or evaluation of detection methods is performed, a reference standard is needed. Frequently, this is achieved by histological examination [3]. This leads to irreversible loss of the teeth.

In order to overcome this problem, teeth can be photographed before sectioning to keep them at least virtually available all by having a reference standard at hand. Development of digital photography has allowed producing photographs of high quality which can be reproduced, viewed and distributed nearly limitless and without loss of quality. This was demonstrated in a study for the histological reference standard but not yet for the occlusal surfaces of the teeth used in that study [4]. In several studies, photographs of teeth or tooth surfaces have been used for the purpose of epidemiology [5], detection of caries [6–8] or enamel disorders [9]. The comparison with a reference standard has shown that sensitivity and specificity obtained on photographs and extracted teeth were comparable [6]. Furthermore, in studies using clinical examinations [10], photographs allow the calibration of multiple observers or in repetition without putting undue stress on the volunteers, especially children [11], however without being able to use a histological reference standard. Comparing caries detection on teeth and photographs has been done before, however not using the full scale of ICDAS scores. Boye et al. [6] used a comparatively simple scoring system for caries for the purpose of epidemiological surveys. The score was the presence or absence of dentine caries. The study showed a good correlation. However, ICDAS, with a more elaborate scale of lesion severities could produce different results. Gomez et al. [12] showed no difference in detection of non-cavitated lesions between teeth or their photographs. In this study, a general agreement was studied using correlation and kappa analysis.

The aim of this study was to evaluate the performance of ICDAS scores obtained on extracted teeth compared to their photographs using histology as a reference standard. The working hypothesis was that there is no statistical difference between the diagnostic performances of both techniques. Furthermore, the effect of observer’s different background and experience was evaluated by using photographs of teeth. The hypothesis was that the training of the examiner has an influence on detection ability. Therefore two other dentists with less experience in cariology research or ICDAS were added to the panel.

## Methods

### Sample selection and preparation

Teeth were selected from a collection extracted for periodontal or orthodontic reasons at University of Dundee and Marburg university dental schools in 2007. Oral consent of the patients was obtained according to the ethical rules of that time. In the meantime, Brussels University medical ethical committee allowed collection of teeth extracted in the framework of routine patient treatment for diagnostic research purposes (ref. B14320096266). 100 unrestored posterior teeth were selected from a batch of extracted and cleaned teeth stored in a thymol solution. Selection was performed based on visual criteria to obtain a number of sound teeth and a range of occlusal carious lesions in different states. Teeth with discoloration not due to carious changes were excluded since this was not a part of the hypothesis under study. Prior to examination or taking of photographs, the tooth surfaces were carefully dried with compressed air. Teeth were then immediately returned to containers with 100 % humidity. High resolution photographs were taken from the occlusal surfaces under standardized conditions with a digital camera and anti-dazzle macro lens (EOS 30 D, MP5; f = 65 mm, Canon Comp, Krefeld, Germany). The teeth were illuminated using an oblique incident light from a ring-shaped fluorescent tube which reproduced daylight. The resolution of the camera chip was 3.5 mega pixels [4]. After performing caries detection using the ICDAS criteria [13], serial sections of the teeth were provided for the establishment of a reference standard and digital images were obtained referring to a published protocol [4]. In brief, one to four histological sections and their images were assigned to each examination site. All images of the selected sections for each investigation site were analyzed by all four examiners using the following histological classification [14]: 0 = No enamel demineralization or narrow surface zone of opacity, 1 = Enamel demineralization limited to the outer 50 % of enamel, 2 = Demineralization involving the inner 50 % of enamel, up to the enamel–dentine junction (EDJ), 3 = Demineralization involving the outer 50 % of dentine, 4 = Demineralization involving the inner 50 % of dentine.

The histological score assigned to each investigation site represented the worst classification observed in the representative sections. Emphasis was placed on differentiating pulp–dentine complex reactions with dental tissues affected by caries. Consensus score between all examiners for each examination site served as reference standard [4].

The teeth were dried and examined by a panel of trained observers using the ICDAS criteria. The sites were recorded as: 0 = sound; 1 = first visible sign of non-cavitated lesion seen only when the tooth is dry; 2 = visible non-cavitated lesion seen when wet and dry; 3 = localized enamel breakdown without visual signs of dentine involvement; Code 4 = underlying dark shadow from dentine with or without localized enamel breakdown; Code 5 = distinct cavity with visible dentine; Code 6 = extensive cavitated lesion with visible dentine [13].

The digital photographs were saved on a CD-ROM and made accessible to 4 examiners. Two of them (AJM and VS) were identical to a panel of observers from a previous study [4], while two other examiners were newly recruited (PB and CB). Three of the observers had research experience in caries detection (AJM, VS and PB), one was last year student from dental school (CB). Of the examiners, 3 received extensive training in ICDAS (AJM, VS, CB) while one used the e-learning program for ICDAS [15] only (PB). Each examiner viewed the digital images on a LCD color monitor at a constant observation distance (60 cm).

Scoring according to the ICDAS criteria was performed on a computer screen without magnification or other image manipulations using a standard image viewing software (IrfanView). A printout at lower resolution indicating the sites to be scored per tooth and a form for the recording of the scores was presented to the examiner. Two consecutive scorings were performed with a four-week interval. The results of an earlier study of ICDAS scores [4] performed by visual inspection on the original extracted teeth (with 2 of the observers of the original 4-person panel, AJM and VS) served as comparison.

### Statistical analysis

In total 169 sites were evaluated. Forty-three teeth had one site, 46 two, 10 teeth three and one tooth had four investigation sites. In order to exclude potential confounding factors by caries extending below the surface to neighboring sites, only one site per tooth was selected after scoring, using computer-generated random numbers. Analysis was performed via sensitivity and specificity (expressed as receiver-operating characteristic (ROC)-curves) and repeatability (expressed as intra-rater kappa). Variability between observers was estimated using Bland-Altman plots and repeated measures ANOVA. In all statistical hypothesis tests, a p-value of 0.05 or lower was chosen as a limit for significance.

Sensitivity and specificity were calculated (cutoff: dentine caries, histology D3 and ICDAS = 3) and ROC curves were established. Statistical comparison between correlated ROC-AUC values of both scoring techniques was performed using the tooth surface (and not the global result of one observer) as unit [16]. Further analysis was performed using kappa statistics between observers (inter-rater) and between both repeat scorings (intra-rater). Intra-rater kappa statistics was also calculated separately for histologically sound teeth and those with enamel or dentine caries. These calculations were performed with the R software (version 2.15.1, The R Foundation for Statistical Computing). Furthermore Bland-Altman-plots were established (Prism version 5.0, GraphPad, LaJolla, USA) between observers scoring photographs and histology as well as between the two observers having scored both teeth and photographs. A repeated measure ANOVA was performed to evaluate the effect of the observer with histological lesion category as covariate. A significant (*p* < 0.001) Mauchly test indicated that the compensation according to Greenhouse-Geiser was to be used for the calculation of within-subject effects. These statistical calculations were performed using SPSS (version 22.0, SPSS inc, Armonk, NY).

## Results

Four teeth were lost during histological preparation, leaving 96 teeth for further evaluation. Of the 96 sites selected, 27 were histologically sound, 16 had shallow and 16 deep enamel caries, 26 had superficial and 11 deep dentine caries.

Correlation coefficients between histology and ICDAS scores varied between 0.55 and 0.71.

Scoring of the original teeth yielded Az values of the ROC curves of 0.9 and 0.89 respectively, scoring of the photographs resulted in Az values ranging from 0.8 to 0.88 (Table 1). There was no significant difference between both modalities (*p* > 0.05).

**Table 1:** Sensitivity and specificity and ROC-AUC (Az) calculated for different cut-off values and modalities

Observer and modality	Cut-off histology = 1, ICDAS = 1		Cut-off histology = 3, ICDAS = 4,		
	Sensitivity	Specificity	A_z_	Sensitivity	Specificity
Obs. 1, tooth	0.94	0.44	0,89	1.00	0.27
Obs. 2, tooth	0.72	0.48	0,9	0.97	0.52
Obs. 1, photo	0.86	0.56	0.88	0.97	0.41
Obs. 2, photo	0.94	0.41	0.84	0.97	0.24
Obs. 3, photo	0.77	0.63	0.80	0.84	0.45
Obs. 4, photo	1.00	0.19	0.84	1.00	0.08

**Fig. 1:**
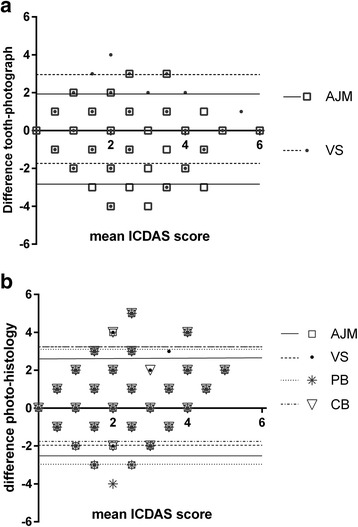
Bland-Altman plot of ICDAS scores of extracted teeth versus their photographs (**a**) and ICDAS scores of photographs versus the histological reference standard (**b**) showing the variation between observers (initials as given in the text). The *lines* show the upper and lower limits of the 95 % CI per observer

Specificity and sensitivity varied, especially in the case of the less experienced observers 3 and 4. The results of the Kappa statistics can be found in Table 2. Kappa values varied according to the histological reference standard of the tooth. Intra-rater kappa values were systematically higher than inter-rater. Values for sound and dentine caries also were higher than for enamel caries. Global Inter-rater (Fleiss) kappa values (all surfaces) were 0.261 for the first scoring and 0.221 for the second scoring (*p* < 0.05). Bland-Altman plots are shown in Fig. 1. The derived parameters (bias and limits of agreement) are given in Table 3.

**Table 2:** Kappa values, inter and intra-rater. O1 to O4: observers. Results are linearly weighted kappa

Modality		Histological state of the surface							
Inter-rater		Sound		Enamel		Dentine		Total	
		k ± S.E.	Sig.	k ± S.E.	Sig.	k ± S.E.	Sig.	k ± S.E.	Sig.
O1-O2	tooth	0.41 ± 0.16	*	0.33 ± 0.10	**	0.65 ± 0.07	***	0.64 ± 0.04	***
O1-O2	photo	0.55 ± 0.12	**	0.26 ± .14	*	0.46 ± 0.10	***	0.57 ± 0.05	***
O1-O3	photo	0.44 ± 0.12	*	0.02 ± 0.14	ns	0.17 ± 0.12	ns	0.39 ± 0.06	***
O1-O4	photo	0.30 ± 0.13	*	0.20 ± 0.13	ns	0.33 ± 0.11	**	0.44 ± 0.05	***
O2-O3	photo	0.53 ± 0.12	**	0.31 ± 0.11	*	0.32 ± 0.12	**	0.52 ± 0.05	***
O2-O4	photo	0.51 ± 0.12	**	0.25 ± 0.10	ns	0.52 ± 0.10	***	0.58 ± 0.05	***
O3-O4	photo	0.40 ± 0.14	**	0.15 ± 0.12	ns	0.26 ± 0.12	*	0.42 ± 0.06	***
Intra-rater		Sound		Enamel		Dentine		total	
O1	tooth	0.82 ± 0.07	***	0.54 ± 0.09	***	0.86 ± 0.06	***	0.85 ± 0.03	***
O2	tooth	0.54 ± 0.12	*	0.49 ± 0.09	**	0.75 ± 0.06	***	0.74 ± 0.04	***
O1	photo	0.62 ± 0.15	**	0.54 ± 0.11	***	0.67 ± 0.10	***	0.74 ± 0.04	***
O2	photo	0.50 ± 0.11	**	0.34 ± 0.14	*	0.53 ± 0.10	***	0.61 ± 0.05	***
O3	photo	0.44 ± 0.16	*	0.29 ± 0.12	*	0.81 ± 0.07	***	0.59 ± 0.05	***
O4	photo	0.82 ± 0.07	***	0.57 ± 0.15	***	0.73 ± 0.06	***	0.78 ± 0.04	***

Significance levels are given as follows: ns: not significant (*p* > 0.05), * *p* < 0.05 > 0.01, **p < 0.01.0.001, ***p < 0.001

**Table 3:** Results of the Bland-Altman test for different modalities and observers. Bias: asymmetric distribution of the discrepancies between the modalities. Limits of agreement: 95 % of the data fall between these limits

Modality	Bias (±SD)	95 % limits of agreement	
		Lower	Upper
Observer 1, tooth vs histo	−0.46 ± 1.30	−3.00	2.08
Obs. 2, tooth vs histo	0.0 ± 1.38	−2.70	2.70
Obs 1, tooth vs photo	0.43 ± 1.19	−1.90	2.75
Obs 2, tooth vs photo	−0.60 ± 1.21	−1.90	2.75
Obs 1, photo vs histo	0.03 ± 1.17	−2.25	2.31
Obs 2, photo vs. histo	0.59 ± 1.31	−1.98	3.16
Obs 3, photo vs histo	0.15 ± 1.54	−2.68	3.15
Obs 4, photo vs histo	0.67 ± 1.24	−1.74	3.10

The results of the repeated measure ANOVA showed that for the observers scoring both teeth and photographs, the variable “operator” (observer 1 or 2) had a F-value of 0.63, *p* = 0.43, “modality” (tooth or photograph) yielded a F-value of 1.57, *p* = 0.21. The two-way interaction effects yielded significant effect (modality*operator F = 40.57, *p* < 0.001) and (modality*histology F = 3.25, *p* = 0.015). In the case of the four observers scoring photographs, observer was the most prominent variable (F = 10.56, *p* < 0.001).

**Fig. 2:**
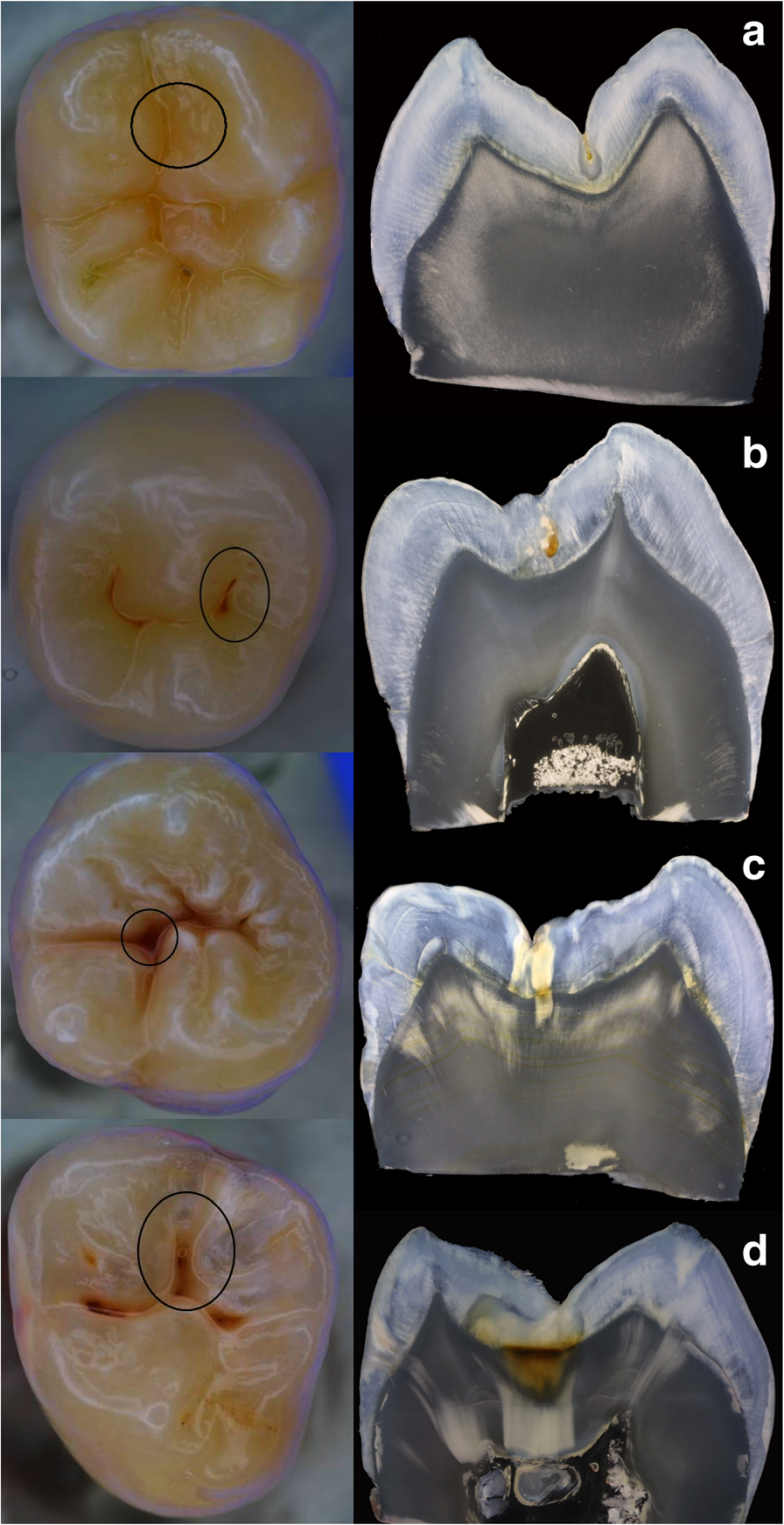
**a**-**d** Examples of different teeth used in the study. The investigation sites are marked with a circle: **a** Example of a tooth being scored as sound while being histologically carious. **b** Tooth with ICDAS score 2 and corresponding histological images. **c** Tooth with ICDAS score 3 and corresponding histological images. **d** Tooth with ICDAS score 4 and corresponding histological images

In Fig. 2, representative samples of teeth and the corresponding histological sections are presented for different ICDAS scores.

Statistical power was calculated using a specific method for ROC analysis [16]. Based on Az values and their standard errors a power ranging from 0.14 to 0.29 for a difference of 5 % between raters and 0.39 to 0.80 for a difference of 10 % in Az value between raters could be calculated. For a difference between methods (tooth vs photograph) power was calculated to range between 0.28 and 0.40 for a 5 % difference between methods and 0.79 to 0.93 for a 10 % difference.

## Discussion

In this in vitro study it could be shown that visual caries detection according to the ICDAS on extracted teeth and their photographs achieved a good agreement and had AUC values in the ROC curves exceeding 0.73 and acceptable to good intra- and inter-rater agreement. Direct observation scored somewhat better (ROC 0.84). The fact that in the direct observation higher (but not significantly so) AUC values were found could be attributed to the high scores they showed in the previous study from which the data were taken [4]. The training and discussion of the observers in the preparation of that study is certainly a major reason. Training and calibration of these two observers may also be the reason that no observer effect was found in the ANOVA for the direct observation of teeth. Nevertheless, in the previously performed study [4] the aim was to evaluate examiner reproducibility in the assessment of caries lesion depth when viewing images of histological sections on a computer monitor compared to direct microscopy.

In the present study in no case, a perfect detection was possible. Consistently, several histologically sound sites were scored as carious and vice versa (Fig. 2). In the case of false-positive scores, discoloration and deep-narrow fissures with stain accumulation were involved. This phenomenon has been described as a “typical pitfall” for ICDAS by Altarakehma et al. [17].

Hintze et al. [18] proposed a minimum quantity of either surfaces/sites or observers in order to reach significant differences between radiographic detection methods. This calculation was based on an experimental design in which the individual observer’s ROC curve is used as variable as unit of statistical calculation. In the present study, calculation and comparison of ROC curves was performed using the tooth as unit resulting in a larger data base for statistical comparisons.

A potential weakness of the experimental design was that the original research team was not more available and teeth were already sectioned. So we were not able to assess the scores of trained examiners on scoring photographs or less experienced observers of scoring the original teeth. With using the surface as unit, however, we believe that the statistical material would have been sufficient to demonstrate a difference with just these two remaining observers.

The power of the present study design was rather low. However, power calculations are intended to be applied for the demonstration of a possible difference, not for equivalence as was the aim of the present study.

The effect of photograph on observer’s performance has already been examined and showed no significant difference in sound surfaces or those with ICDAS scores 1–4 [12]. With our data, we could show the same for the whole range of ICDAS scores. What seemed more important however, was the disparity between observers. Observer 4 had a very high sensitivity but a low specificity, notwithstanding the cut-off value used (Table 1).

Recently, Altarakehma et al. [17] and Qudeimat el al. [19] stressed the importance of the contribution of the individual observer to the result. Using photographs would allow to further explore this hypothesis as the material can be made accessible to larger groups of observers also by using digital media. Thus, studies would be less dependent on logistical aspects [8]. This is beneficial for multi-centre studies [4], teaching purposes and it also helps increasing the number of participants for such type of studies and hence to achieve valid power for interpreting the data.

Another interesting finding is that inter- and intra-rater kappa values varied according to the histological state of the tooth (Table 2). Intra-rater values were lowest for ICDAS 1–3 lesions. The same applied for inter-rater. The kappa values were close to nothing when the inexperienced observers (3 and 4) were matched against the experienced ones. The same was reflected in the Bland-Altman plots and analysis. This was in contrast to the findings of Diniz et al., [20] and may have been a result of a different balance between histological grades. The advantage of using photographs would be in this context that a balance can be made between histological grades before undertaking the experiment and letting the observers score the selected photographs. It would also allow studying the effect of different balances of histological gradation, for instance such as in a given population and compare this to a selection favoring a numerical balance.

Only very few teeth were scored differently to histology in only one modality. This is reflected in the small difference in ROC curve AUC for both methods. Photographs, although not (yet) being able to give a three-dimensional image, seem to be suitable for caries detection [6]. Also in other domains such as dental material evaluation, standardized photographs seem to be valuable tools of observation [21]. Standardization is important if texture and color (as used in the ICDAS definition) has to be judged. However the method used in this study did not use an elaborate color standardization protocol as the one described by Bengel [22] for the evaluation of bleaching techniques. It was deemed that strict color standardization was not needed because no consecutive evaluation of color change was intended.

One shortcoming of using photographs is the fact that the ICDAS scores should be determined after air drying the tooth surface. Hence in the present study proper differentiation of scores ICDAS1 and ICDAS 2 would not be possible. The question whether there is a benefit of using pictures of wet and dry tooth surfaces for detection of initial lesions on digital images was addressed by Jablonski-Momeni et al. [23, 24]. The authors showed that the diagnostic performance of the examinations (AUC) was consistent with a good to very good diagnostic performance irrespective of whether the surface was photographed wet or dry [23]. There was no considerable difference in the diagnostic accuracy of the ICDAS when the performance was evaluated either on images of wet or dried tooth surfaces [24]. In a recently published manual there are also suggestions to merge the ICDAS codes for the purpose of caries management [25]. Here the ICDAS codes 1 and 2 are categorized as initial stage of caries and are explained as “First or distinct visual changes in enamel seen as a carious opacity or visible discoloration (white spot lesion and/or brown carious discoloration) not consistent with clinical appearance of sound enamel (ICDAS code 1 or 2) and which show no evidence of surface breakdown or underlying dentine shadowing”. Specifically for pits and fissures “the carious discoloration is apparent starting in the base of the fissure or pit and may extend up the wall of the pit/fissure but no distinct loss of enamel is apparent, i.e. the pit/fissure retains its original anatomical appearance. Appearance not consistent with stained pits/fissures (ICDAS code 0)”. Hence the differentiation of initial and moderate lesions might still be possible when tooth images are used. The effect of grey value vision has been forwarded as possible source of bias in caries detection on radiographs [26] and it remains to be demonstrated if a similar phenomenon pertaining to color perception may have played a role in this study. Notwithstanding which method is used, caries detection methods rely upon the human eye and mind as tool for interpretation of mainly visual signs. In future studies a higher number of observers with a different background should be used for data generation in order to obtain a realistic appreciation of a method. Fortunately with the present data and also based on the study of Gomez et al. [12], photographs can be advantageously used to increase the number of observers. This hypothesis will be the object of future continuation of this research work.

## Conclusions

The modality of scoring ICDAS (original teeth or their digital photographs) differs less than the observers among each other. Using photographs can facilitate the logistic aspect of multi-center studies and increase the number of observers next to being useful in clinical training and teaching.

Parts of this manuscript have been presented as a poster on the ORCA conference in Liverpool, 2013 (abstract nr. 92).

## Abbreviations

ANOVA: Analysis of variance
AUC: Area under the curve
ICDAS: International Caries Detection and Assessment System
ROC: Receiver-operating characteristics
